# Knowledge, Attitudes, and Practices of Intern Doctors Regarding the Use of Inhalation Devices at a Tertiary Care Teaching Rural Hospital

**DOI:** 10.7759/cureus.115824

**Published:** 2026-09-05

**Authors:** Archana Patel, Rujuta Sanghvi

**Affiliations:** 1 Department of Pharmacology, Sumandeep Vidyapeeth, Vadodara, IND

**Keywords:** dry powder inhaler (dpi), inhalation therapy, knowledge attitude practice (kap), medical interns, metered-dose inhaler (mdi), pulmonology education, spacer device

## Abstract

Background: Inhalation therapy is the cornerstone of management for chronic obstructive pulmonary disease and bronchial asthma, offering rapid therapeutic onset, lower dosage requirements, and fewer systemic adverse effects than oral formulations. However, the clinical efficacy of aerosolized pharmacotherapy depends heavily on proper inhalation technique. As frontline healthcare providers frequently tasked with patient counseling and device demonstration, medical interns play a pivotal role in ensuring proper device utilization. This study aimed to evaluate the knowledge, attitudes, and practical competencies regarding metered-dose inhalers (MDIs), dry powder inhalers (DPIs), and spacer devices among medical interns at a rural tertiary care teaching hospital.

Methods: A questionnaire-based, cross-sectional observational study was conducted among medical interns at Smt. B.K. Shah Medical Institute and Research Centre following Institutional Ethics Committee approval. A prevalidated, structured questionnaire evaluating theoretical knowledge, clinical awareness, environmental considerations, and practical demonstration skills regarding MDIs, DPIs, and spacer devices was administered. Descriptive statistical analysis was performed using frequencies and percentages.

Results: A total of 139 completed questionnaires out of 146 distributed were analyzed, yielding a response rate of 95.2%. Overall, theoretical knowledge was high: 126 (90.6%) participants recognized the correct procedural sequence for MDIs, 135 (97.1%) correctly identified common patient errors with MDIs, and 125 (89.9%) and 129 (92.8%) demonstrated accurate knowledge regarding DPI technique and common DPI errors, respectively. Furthermore, 126 (90.6%) understood the comparative environmental footprints of MDIs vs. DPIs, and 131 (94.2%) were aware of potential adverse effects. However, a notable discrepancy was observed in clinical practice: only 103 (74.1%) had demonstrated MDI technique to patients, with even lower demonstration rates for DPIs (n = 98, 70.5%) and spacers (n = 97, 69.8%).

Conclusions: Although theoretical comprehension of inhalation devices was high among intern doctors, substantial deficits persist in practical demonstration and bedside patient education. Incorporating structured, hands-on workshops, Objective Structured Clinical Examinations, and dedicated inhalation-technique modules into undergraduate medical curricula is imperative to bridge the theory-practice gap, optimize rational prescribing, and improve clinical outcomes in obstructive airway diseases.

## Introduction

Bronchial asthma and chronic obstructive pulmonary disease (COPD) represent major global burdens of chronic respiratory morbidity and mortality [1-3]. Asthma is characterized by variable airway hyperresponsiveness and reversible airflow obstruction, whereas COPD is marked by persistent, progressive, and largely irreversible airflow limitation [1-3]. Modern therapeutic guidelines position targeted inhalation therapy as the cornerstone for managing both conditions, offering rapid clinical onset, targeted topical pulmonary delivery, lower required doses, and substantially reduced systemic adverse events compared with oral formulations [3-5]. The clinical efficacy of aerosol pharmacotherapy relies fundamentally on the patient's device mastery and correct inhalation technique, with pressurized metered-dose inhalers (pMDIs), dry powder inhalers (DPIs), and spacer devices representing the most widely prescribed inhalation modalities [1,4,5].

However, each delivery platform presents distinct operational demands, operational mechanisms, and clinical considerations. pMDIs require precise hand-breath coordination, a deep and slow inhalation initiated from functional residual capacity, and a subsequent 5-10-second breath hold. Due to these complex demands, faulty actuation-inhalation coordination remains the most frequent patient error [6]. Additionally, conventional pMDIs utilize hydrofluoroalkane (HFA) propellants, which have a substantially higher global warming potential than propellant-free alternatives [7]. To overcome these coordination challenges, breath-actuated DPIs, such as the Rotahaler (Cipla Ltd., Mumbai, India), Diskus (GlaxoSmithKline (GSK), Research Triangle Park, NC), and Revolizer (Cipla Ltd., Mumbai, India), were developed. While these devices do not rely on propellants or manual timing of actuation, they require the patient to generate a sufficient inspiratory flow rate to effectively disaggregate the dry powder formulation into respirable fractions [1,8-11].

Spacer devices and valved holding chambers (VHCs) serve as vital adjuncts to pMDIs by bridging the gap between device mechanics and patient capability. By eliminating coordination barriers, spacers reduce oropharyngeal drug impaction and significantly enhance pulmonary deposition, making them particularly valuable for pediatric, geriatric, and cognitively impaired populations [12-14]. Despite these established benefits, faulty inhalation technique remains widespread across outpatient and inpatient settings, directly contributing to subtherapeutic drug delivery, frequent disease exacerbations, and overall treatment failure [10,14,15]. Passive educational strategies such as reading package inserts or viewing instructional videos have proven inferior to direct, provider-led physical demonstrations accompanied by the "teach-back" method [16-18].

Because medical interns serve as frontline clinical staff who frequently prescribe, dispense initial counseling, and demonstrate inhalation equipment at the bedside, their practical competence directly governs patient technique and long-term respiratory outcomes [6,15,18]. Nonetheless, published literature across diverse clinical settings suggests substantial deficits in device proficiency among trainee physicians [4,6,18]. Therefore, this study aimed to evaluate the baseline knowledge, attitudes, and practical competencies regarding MDIs, DPIs, and spacer devices among medical intern doctors at a rural tertiary care teaching hospital.

Primary objective

The primary objective is to evaluate the baseline knowledge, attitudes, and clinical practices (KAP) regarding the use of inhalation devices, specifically pMDIs, DPIs, and spacer devices, among medical intern doctors at a rural tertiary care teaching hospital.

Secondary objectives

The secondary objective is to identify specific knowledge deficits and technical gaps in the step-by-step demonstration of MDIs, DPIs, and spacer devices; to assess interns' awareness of critical device-specific factors, including common patient errors, adverse effects, and comparative environmental impacts (e.g., propellant-driven MDIs vs. breath-actuated DPIs); and to determine the primary sources of clinical education, formal training, and instructional resources utilized by intern doctors concerning inhalation therapy.

## Materials and methods

Study design and setting

A cross-sectional, questionnaire-based descriptive study was conducted over a four-month period from October 2025 to January 2026 at Dhiraj Hospital, a rural tertiary care teaching hospital affiliated with the Smt. B.K. Shah Medical Institute and Research Centre (SBKS MI and RC), Sumandeep Vidyapeeth (Deemed to be University), located in Piparia, Vadodara, Gujarat, India.

Ethical considerations

Prior to study initiation, formal ethical clearance was obtained from the Institutional Ethics Committee (IEC) of Sumandeep Vidyapeeth (IEC Registration Number: EC-CT-2019-0079; Protocol Reference: 25/40; dated: September 26, 2025). All participants were informed of the study objectives, the voluntary nature of participation, and data confidentiality protocols. Digital informed consent was obtained from each respondent prior to access to the questionnaire. Data collection was entirely anonymous, with no personally identifiable information recorded.

Study population and eligibility criteria

The study population comprised medical intern doctors undergoing their compulsory rotational residential internship at SBKS MI and RC and Dhiraj Hospital. The inclusion criteria encompassed all registered medical intern doctors actively posted across clinical departments who provided voluntary digital informed consent. The exclusion criteria comprised intern doctors who declined participation or submitted incomplete survey responses.

Data collection tool and administration

Data were collected using a structured, prevalidated questionnaire designed to evaluate knowledge, attitudes, and practical competencies concerning inhalation devices, including pMDIs, DPIs, and spacer devices (see the Appendix). The survey instrument evaluated four distinct domains: sociodemographic and training characteristics, knowledge, attitudes, and clinical practices regarding inhalation devices. The sociodemographic and training profile captured participants' prior clinical exposure, formal teaching received, and primary learning sources. The knowledge domain assessed understanding of device mechanisms, drug-delivery principles, step-by-step procedural sequences, common patient errors, adverse-effect profiles, and comparative environmental impacts, such as the carbon footprints of propellant-based pMDIs vs. DPIs. The attitude domain explored perceptions regarding the physician's responsibility in patient counseling, the clinical value of technique mastery, and receptiveness to dedicated hands-on training. Finally, the practice domain examined real-world clinical behaviors, specifically focusing on the frequency of direct physical demonstration of metered-dose inhalers (MDIs), DPIs, and spacer devices, alongside routine assessment of patient inhalation technique. The survey was digitized using Google Forms (Google LLC, Mountain View, CA) and distributed electronically via institutional intern communication groups to ensure accessibility and maximize response rates.

Sample size and statistical analysis

Instead of using a sample size formula, a census method was used to include all eligible interns available during the study period. The study included all 146 medical interns enrolled at SBKS MI and RC and Dhiraj Hospital during the study period. An online survey link was sent to every intern. Of the 146 interns invited, 139 completed the questionnaire, yielding a high response rate of 95.2% (n = 139). Seven interns did not complete or submit the survey. With 139 of 146 interns participating, this sample has a margin of error of less than 3% at the 95% confidence level. All responses were extracted, coded, and entered into Microsoft Excel (Microsoft Corp., Redmond, WA). Descriptive statistics, including frequencies and percentages, were computed for categorical variables to summarize the knowledge, attitude, and practice profiles of the study cohort.

## Results

A total of 139 completed questionnaires out of 146 distributed were analyzed, yielding a response rate of 95.2%. Among the 139 participating intern doctors, female interns predominated, comprising 77 (55.4%) respondents, while male interns accounted for 62 (44.6%) of the study (Table 1).

**Table 1:** Baseline demographic profile of the study participants (n =139)

Demographic variable	Values, n (%)
Gender	
Female	77 (55.4%)
Male	62 (44.6%)
Total analyzed	139 (100.0%)

Overall theoretical knowledge across different inhalation devices was consistently high among respondents (Table 2). Regarding pMDIs, 126 (90.6%) interns correctly identified the full expansion, procedural steps, and environmental consequences associated with propellant gases, while 135 (97.1%) recognized the most common operational errors committed by patients during MDI actuation.

**Table 2:** Assessment of theoretical knowledge regarding MDIs, DPIs, and spacer devices (n = 139) MDI: metered-dose inhaler; DPI: dry powder inhaler

Knowledge assessment parameter	Device/aspect	Correct responses, n (%)
Full form, correct operational sequence, and environmental impact	MDI	126 (90.6%)
Identification of most common patient errors	MDI	135 (97.1%)
Requirement of rapid and deep inhalation for drug delivery	DPI	129 (92.8%)
Correct postloading inhalation technique	DPI	125 (89.9%)
Recognition of common operational errors	DPI	129 (92.8%)
Requirement of adequate inspiratory effort	DPI	127 (91.4%)
Lower environmental impact/eco-friendly profile compared to MDIs	DPI	126 (90.6%)
Indications, functions, and proper use of spacers	Spacer	130 (93.5%)
Complications and clinical risks of faulty inhalation technique	General	131 (94.2%)

With respect to DPIs, 129 (92.8%) interns demonstrated a correct understanding of drug-delivery mechanics requiring rapid, deep inhalation, and 125 (89.9%) accurately identified the sequential steps following dose loading. Awareness of common DPI errors was observed in 129 (92.8%) respondents; 127 (91.4%) recognized that DPIs require a forceful inspiratory effort; and 126 (90.6%) understood that DPIs have a lower environmental carbon footprint than MDIs.

Additionally, 130 (93.5%) participants demonstrated adequate knowledge regarding the indications and functions of spacer devices, and 131 (94.2%) were aware of the complications and adverse effects stemming from faulty inhalation technique.

Attitudes toward patient education and professional development were overwhelmingly favorable (Table 3). A total of 127 (91.4%) interns strongly affirmed that proper inhalation technique is critical for effective clinical disease control and acknowledged that patient counseling represents an essential physician responsibility. Furthermore, 124 (89.2%) interns strongly agreed that structured, hands-on training programs would substantially enhance their technical skills and clinical competence.

**Table 3:** Attitudes of intern doctors toward inhalation device counseling and training (n = 139)

Attitude parameter	Strongly agree, n (%)	Agree, n (%)	Neutral, n (%)
Proper technique is essential for effective disease management	127 (91.4%)	8 (5.8%)	4 (2.9%)
Educating patients on device technique is a core physician responsibility	127 (91.4%)	10 (7.2%)	2 (1.4%)
Structured training would improve practical skills and clinical performance	124 (89.2%)	2 (1.4%)	13 (9.4%)

In clinical practice, 103 (74.1%) interns reported having actively demonstrated inhalation devices to patients at the bedside (Table 4). When evaluating device demonstration proficiency across the entire cohort, 103 (74.1%) participants correctly demonstrated MDI technique, 98 (70.5%) DPI technique, and 97 (69.8%) spacer use.

**Table 4:** Clinical demonstration practices and device proficiency among intern doctors (n = 139) MDI: metered-dose inhaler; DPI: dry powder inhaler

Device demonstration parameter	Number of interns, n (%)
Interns who have demonstrated inhaler use to patients	103 (74.1%)
Correct demonstration of MDI	103 (74.1%)
Correct demonstration of DPI	98 (70.5%)
Correct demonstration of spacer	97 (69.8%)

## Discussion

Optimal pharmacotherapeutic management of asthma and COPD hinges on accurate, patient-centered drug delivery. Although a wide array of advanced inhalation devices is clinically available, their therapeutic efficacy fundamentally depends on the ability of healthcare professionals to correctly educate, evaluate, and demonstrate device technique to patients [19]. Frontline physicians, particularly medical interns during their foundational clinical rotations, are routinely tasked with selecting appropriate delivery platforms and delivering effective bedside instruction [6].

In the present study, medical interns demonstrated commendable theoretical knowledge across various inhalation platforms. A substantial majority accurately identified the sequential steps for pMDI administration and recognized hand-breath dyssynchrony as the most prevalent patient error [6]. Furthermore, participants exhibited high awareness of DPIs, notably recognizing the need for a forceful, rapid inspiratory effort to achieve adequate drug micronization, as well as the utility of VHCs (spacers) in overcoming coordination barriers in vulnerable patient subsets. Respondents were also cognizant of local adverse effects, correctly identifying oropharyngeal candidiasis as a common complication that can be effectively mitigated through postinhalation mouth rinsing. Notably, over 90% of participants recognized the higher carbon footprint associated with HFA propellants in MDIs compared with propellant-free DPIs, highlighting encouraging awareness of sustainable, environmentally conscious respiratory prescribing practices [7].

Attitudes toward patient education and professional development were overwhelmingly positive. More than 91% of respondents affirmed that patient counseling is a fundamental responsibility of physicians and acknowledged that optimal inhalation technique is essential for favorable clinical outcomes. Additionally, nearly 90% supported integrating formal hands-on training workshops into their clinical training.

Despite robust cognitive competence, a notable divergence between theoretical knowledge and real-world clinical application emerged. While theoretical understanding exceeded 90% across most domains, only 74.1% of interns reported having physically demonstrated the MDI technique to patients, with even lower practical demonstration rates observed for DPIs (70.5%) and spacer devices (69.8%). This theory-practice gap is consistent with observations by Maepa et al., who noted that high theoretical awareness among healthcare trainees does not reliably translate into routine bedside demonstrations during patient encounters [20]. Similarly, Kshatriya et al. reported that trainee physicians frequently encountered difficulties with physical device manipulation despite possessing baseline theoretical knowledge [6].

Several systemic factors likely drive this implementation deficit. Undergraduate medical curricula and internship training structures traditionally prioritize acute emergency management and diagnostic reasoning, often leaving little room for structured, supervised instruction on device mechanics. Moreover, high patient volumes and stringent time constraints in outpatient settings frequently disincentivize comprehensive "teach-back" demonstrations.

Addressing this gap will require targeted curricular and institutional reforms. Incorporating interactive hands-on workshops, Objective Structured Clinical Examinations (OSCEs), and simulated counseling sessions into undergraduate and intern orientation programs can solidify practical mastery [16,18]. At the institutional level, equipping outpatient departments and clinical wards with placebo training devices and standardized instructional checklists can empower early-career physicians to routinely and confidently perform physical demonstrations.

Limitations

Several limitations of this study warrant consideration. First, this investigation was conducted at a single rural tertiary care teaching hospital, which may limit the generalizability of the findings to institutions with alternative pedagogical frameworks. Second, practical demonstration rates and clinical competence were assessed via self-reported questionnaire responses rather than through direct, real-time observational scoring against validated checklists. Future multicenter studies utilizing objective physical skill assessments before and after structured educational interventions are recommended.

## Conclusions

Medical interns in this study demonstrated strong theoretical knowledge, positive attitudes toward patient counseling, and commendable awareness of environmental sustainability in respiratory pharmacotherapy. However, a distinct theory-practice gap remains, with practical bedside demonstration rates lagging substantially behind cognitive understanding. Addressing this implementation deficit requires integrating mandatory, simulation-based workshops, objective skill assessments (such as OSCEs), and accessible placebo demonstration devices into clinical training. Empowering frontline trainee physicians with practical device mastery is essential to optimizing patient education, enhancing inhaler adherence, and improving clinical outcomes in asthma and COPD management.

## Appendices

**Table 5:** Questions for intern's knowledge, attitude, and practice for the uses of inhalation devices pMDI: pressurized metered-dose inhaler; COPD: chronic obstructive pulmonary disease

Item no.	Question/prompt	Response options
1	Gender	Male
		Female
		Other
2	Duration of internship (in months)	Open numerical entry (months)
3	Have you received formal training in the use of inhalation devices?	Yes
		No
4	Have you demonstrated the use of inhalers (MDI/DPI/Spacer) to any patient during internship?	Yes
		No
5	If yes, then how many numbers of patients have you demonstrated?	Open numerical entry (number of patients)
Knowledge section		
6	What does MDI stand for?	Metered-Dose Inhaler
		Multiple Drug Inhaler
		Medium-Duration Inhaler
		None of the above
7	What is the correct sequence of steps when using a pMDI? (Select the correct order)	Remove cap → Shake inhaler → Breathe out → Press inhaler and inhale slowly (synchronise) → Hold breath for 10 seconds
		Shake inhaler → Breathe in → Remove cap → Inhale deeply
		Press inhaler → Remove cap → Shake inhaler
8	What is the most common mistake made by patients when using a pMDI?	Not shaking the inhaler
		Failing to coordinate inhalation and actuation
		Breathing too deeply
		Cleaning the inhaler too often
9	Does the use of Metered-Dose Inhalers (MDIs) have an impact on the environment?	Yes
		No
		Not sure
10	If yes then, what type of gas used in MDIs contributes to environmental pollution?	Carbon dioxide (CO_2_)
		Hydrofluorocarbons (HFCs)
		Methane
		Nitrogen
11	Which of the following devices is a Dry Powder Inhaler (DPI)? (Select all that apply)	Rotahaler
		Diskus
		Revolizer
		Nebulizer
12	What is required for proper drug delivery in DPI?	Rapid and deep inhalation
		Slow and shallow inhalation
		Breath-hold before inhalation
		Use of spacer
13	Do DPIs require shaking before use?	Yes
		No
14	What is the correct technique for using a DPI?	Shake the inhaler and inhale slowly
		Inhale rapidly and deeply after loading the dose
		Inhale slowly and hold your breath before inhaling
		No specific technique is needed
15	Do DPIs require hand-breath coordination like MDIs?	Yes
		No
		Sometimes
		Not sure
16	Which of the following errors is common with Dry Powder Inhalers (DPIs)?	Inhaling too slowly
		Using a spacer with a DPI
		Not sealing lips around the mouthpiece properly
		All of the above
17	Do Dry Powder Inhalers (DPIs) have a lower environmental impact compared to MDIs?	Yes
		No
		Not sure
18	Why are DPIs considered more environmentally friendly than MDIs?	They contain fewer chemicals
		They use breath-actuation and do not require propellant gases like hydrofluorocarbons (HFCs)
		They produce less medical waste
		All of the above
19	Are propellant-free DPIs being promoted globally as a greener alternative to MDIs?	Yes
		No
		Not sure
20	Which of the following inhalers is considered more environmentally friendly?	MDI (Metered-Dose Inhaler)
		DPI (Dry Powder Inhaler)
		Nebulizer
		All are equally eco-friendly
21	For effective delivery of medication, a DPI requires:	Strong inspiratory effort
		Use of a spacer
		A nebulizer adapter
		Slow, shallow breathing
22	Which group of patients may have difficulty using a DPI?	Children under 5 years
		Elderly patients with weak inspiratory flow
		Patients with severe COPD or neuromuscular disorders
		All of the above
23	In which of the following patient groups is DPI most suitable?	Adults with good inspiratory flow
		Bedridden ICU patients
		Infants and toddlers
		Unconscious patients
24	What is the function of a spacer when used with an MDI?	It reduces the dose
		It slows down the medication and allows easier inhalation
		It increases side effects
		It cleans the medicine
25	Which of the following patients benefit most from using a spacer?	Elderly patients
		Children under 5 years
		Patients with coordination difficulties
		All of the above
26	Does using a spacer reduce oropharyngeal deposition of inhaled corticosteroids (ICS)?	Yes
		No
27	Which of the following is a likely result of incorrect inhaler use?	Reduced effectiveness of the medication
		Frequent disease exacerbations
		Increased hospital visits and healthcare costs
		All of the above
28	Which of the following is a common oral complication associated with inhaled corticosteroids?	Gingivitis
		Oral candidiasis
		Dental caries
		None of the above
29	Do you know the recommended oral hygiene practice after using an inhaler?	No action needed
		Rinse mouth with water
		Brush teeth immediately
		Gargle with mouthwash only
Attitude section (5-Point Likert Scale)		
30	I believe correct inhaler technique is essential for effective asthma/COPD management.	Strongly Disagree/Disagree/Neutral/Agree/Strongly Agree
31	It is moral responsibility to educate patients on MDI and DPI use.	Strongly Disagree/Disagree/Neutral/Agree/Strongly Agree
32	I feel confident in demonstrating MDI, DPI, and spacer use to patients.	Strongly Disagree/Disagree/Neutral/Agree/Strongly Agree
33	Spacer devices are only useful for pediatric patients.	Strongly Disagree/Disagree/Neutral/Agree/Strongly Agree
34	DPI devices are easier for patients to use than MDIs.	Strongly Disagree/Disagree/Neutral/Agree/Strongly Agree
35	Additional hands-on training would improve my skills in using inhalation devices.	Strongly Disagree/Disagree/Neutral/Agree/Strongly Agree
Practice section		
36	Have you ever demonstrated correct MDI technique to a patient?	Yes
		No
37	If yes, then how many numbers of patients have you demonstrated?	Open numerical entry (number of patients)
38	Have you ever demonstrated correct DPI technique to a patient?	Yes
		No
39	If yes, then how many numbers of patients have you demonstrated?	Open numerical entry (number of patients)
40	Have you ever demonstrated how to use a spacer with an MDI?	Yes
		No
41	If yes, then how many numbers of patients have you demonstrated?	Open numerical entry (number of patients)
42	Do you check whether patients know how to use their inhalers during rounds?	Always
		Sometimes
		Rarely
		Never
43	Do you assess a patient’s inspiratory flow capacity before recommending a DPI?	Always
		Sometimes
		Rarely
		Never
44	Which devices have you handled during your internship? (Select all that apply)	pMDI
		DPI (Rotahaler, Diskus, Revolizer)
		Spacer
		Nebulizer
45	Have you observed patients using inhalers incorrectly during your internship?	Yes
		No
46	If yes, what type of errors did you notice?	Not shaking the MDI
		Not holding breath after inhalation
		Poor coordination
		Inhaling too weakly with a DPI
		Others (Open text entry)
47	When you see a patient using an inhaler incorrectly, do you take time to correct their technique?	Always
		Sometimes
		Rarely
		Never
48	Which of the following do you think is the most effective method to improve your knowledge and skills on inhaler devices?	Hands-on training or workshops
		Watching video demonstrations
		Reading instruction leaflets/manuals
		Attending lectures or seminars
		Peer learning or case-based discussions
49	Have you ever used any of the following to improve your inhaler technique knowledge? (Select all that apply)	Faculty-led demonstration
		YouTube or online videos
		Self-practice using a placebo device
		Mobile apps or online quizzes
		I have not used any method yet
50	Have you received any practical or bedside training on inhaler technique during your internship?	Yes
		No

## References

[1] Baral MA (2019). Knowledge and practice of dry powder inhalation among patients with chronic obstructive pulmonary disease in a regional hospital, Nepal. Int J Gen Med.

[2] Tu Y, Yao Q, Chen Y (2025). Knowledge, attitudes, and practices of inhalation device use among patients with chronic obstructive pulmonary disease. Sci Rep.

[3] Agarwal R, Dhooria S, Aggarwal AN (2015). Guidelines for diagnosis and management of bronchial asthma: Joint ICS/NCCP (I) recommendations. Lung India.

[4] Hanania NA, Wittman R, Kesten S, Chapman KR (1994). Medical personnel's knowledge of and ability to use inhaling devices. Metered-dose inhalers, spacing chambers, and breath-actuated dry powder inhalers. Chest.

[5] Sharma S, Chhabra G, Luhadia SK (2017). Knowledge, attitude, practices of inhalational therapy among nursing staff posted at tertiary care teaching hospital. Int J Res Med Sci.

[6] Kshatriya RM, Khara NV, Paliwal RP, Patel SN (2016). Evaluation of proficiency in using different inhaler devices among intern doctors. J Family Med Prim Care.

[7] Rabin AS, Seelye SM, Weinstein JB (2025). Budesonide-formoterol metered-dose inhaler vs fluticasone-salmeterol dry-powder inhaler. JAMA Intern Med.

[8] Hussain FN, Paravattil B (2020). Assessment of educational inhaler technique interventions among community pharmacists: a systematic review. Integr Pharm Res Pract.

[9] Barrons R, Pegram A, Borries A (2011). Inhaler device selection: special considerations in elderly patients with chronic obstructive pulmonary disease. Am J Health Syst Pharm.

[10] Melani AS, Bonavia M, Cilenti V (2011). Inhaler mishandling remains common in real life and is associated with reduced disease control. Respir Med.

[11] Dhoble S, Kapse A, Ghegade V, Chogale M, Ghodake V, Patravale V, Vora LK (2024). Design, development, and technical considerations for dry powder inhaler devices. Drug Discov Today.

[12] Volerman A, Balachandran U, Siros M, Akel M, Press VG (2021). Mask use with spacers/valved holding chambers and metered dose inhalers among children with asthma. Ann Am Thorac Soc.

[13] Newman SP (2004). Spacer devices for metered dose inhalers. Clin Pharmacokinet.

[14] Barbara S, Kritikos V, Bosnic-Anticevich S (2017). Inhaler technique: does age matter? A systematic review. Eur Respir Rev.

[15] Gaikwad MM, Dabhade SS, Jagtap RP, Ghongane BB (2016). A survey of knowledge of interns about the instructions given to the patients while prescribing various dosage forms. Med J DY Patil Univ.

[16] Puah SH, Goh CY, Chan CL, Teoh AK, Zhang H, Shen Z, Neo LP (2022). Mobile device: a useful tool to teach inhaler devices to healthcare professionals. BMC Med Educ.

[17] Giraud V, Allaert FA, Roche N (2011). Inhaler technique and asthma: feasability and acceptability of training by pharmacists. Respir Med.

[18] Mullerpattan JB, Udwadia ZZ, Kathar SS, Shah HD, Rastogi SA, Pandey KV, Udwadia ZF (2016). Who will teach the teachers: an analysis of the inhaler technique of Indian patients and health care providers in a tertiary health care centre. Lung India.

[19] Abdulameer SA (2018). Knowledge and pharmaceutical care practice regarding inhaled therapy among registered and unregistered pharmacists: an urgent need for a patient-oriented health care educational program in Iraq. Int J Chron Obstruct Pulmon Dis.

[20] Maepa HM, Wong ML, Menezes CN (2019). Evaluation of the knowledge and correct use of metered-dose inhalers by healthcare professionals and medical students in Gauteng Province. Afr J Thorac Crit Care Med.

